# Risk scoring systems for adults admitted to the emergency department: a systematic review

**DOI:** 10.1186/1757-7241-18-8

**Published:** 2010-02-11

**Authors:** Mikkel Brabrand, Lars Folkestad, Nicola Groes Clausen, Torben Knudsen, Jesper Hallas

**Affiliations:** 1 Department of Medicine, Sydvestjysk Sygehus, Esbjerg, Denmark; 2 Department of Anesthesiology, Sygehus Lillebælt, Kolding, Denmark; 3 Department of Clinical Pharmacology, Odense University Hospital, Denmark

## Abstract

**Background:**

Patients referred to a medical admission unit (MAU) represent a broad spectrum of disease severity. In the interest of allocating resources to those who might potentially benefit most from clinical interventions, several scoring systems have been proposed as a triaging tool.

Even though most scoring systems are not meant to be used on an individual level, they can support the more inexperienced doctors and nurses in assessing the risk of deterioration of their patients.

We therefore performed a systematic review on the level of evidence of literature on scoring systems developed or validated in the MAU. We hypothesized that existing scoring systems would have a low level of evidence and only few systems would have been externally validated.

**Methods:**

We conducted a systematic search using Medline, EMBASE and the Cochrane Library, according to the PRISMA guidelines, on scoring systems developed to assess medical patients at admission.

The primary endpoints were in-hospital mortality or transfer to the intensive care unit. Studies derived for only a single or few diagnoses were excluded.

The ability to identify patients at risk (discriminatory power) and agreement between observed and predicted outcome (calibration) along with the method of derivation and validation (application on a new cohort) were extracted.

**Results:**

We identified 1,655 articles. Thirty were selected for further review and 10 were included in this review.

Eight systems used vital signs as variables and two relied mostly on blood tests.

Nine systems were derived using regression analysis and eight included patients admitted to a MAU. Six systems used in-hospital mortality as their primary endpoint.

Discriminatory power was specified for eight of the scoring systems and was acceptable or better in five of these. The calibration was only specified for four scoring systems. In none of the studies impact analysis or inter-observer reliability were analyzed.

None of the systems reached the highest level of evidence.

**Conclusions:**

None of the 10 scoring systems presented in this article are perfect and all have their weaknesses. More research is needed before the use of scoring systems can be fully implemented to the risk assessment of acutely admitted medical patients.

## Background

Patients referred to a medical admission unit (MAU) represent a broad spectrum of disease severity. In the interest of allocating resources to those who might potentially benefit most from clinical interventions, several scoring systems have been proposed as a triaging tool.

McClish et al[1] has shown that in a critical care environment, physicians outperform scoring systems when assessing groups of patients at the extremes of risk of deterioration. Patients doing very poorly or very well are easily identified, but when assessing the in-between group scoring, systems were better than clinical experience.

Apart from the assessment of patient risk, scoring systems can be used in clinical trials to account for the severity of disease in the subjects included in the trial, or to adjust for case-mix when benchmarking the performance of clinical units. Finally, they can be used to monitor the effect of new technology. Most systems are not developed to be used on an individual level but on groups of patients.

The development of scoring systems began in the intensive care environment (ICU). Systems such as Acute Physiology and Chronic Health Evaluation (APACHE)[2], the Mortality Probability Models (MPM)[3] and Sequential Organ Failure Assessment (SOFA)[4] scores were developed and validated in ICU's. Later, the Emergency Medicine community caught on and scoring systems for this environment were developed.

Even though most scoring systems are not meant to be used on an individual level, they sometimes will be used by more inexperienced doctors and nurses in assessing the risk of deterioration of their patients. To clarify the level of evidence in this field, we therefore decided to perform a systematic review of the literature on scoring systems developed or validated in the MAU. We hypothesized that the existing scoring systems would have a low level of evidence and that only few systems existed that had been externally validated.

## Materials and methods

Our protocol (available upon request from the authors) asked for inclusion of all clinical studies concerning adult medical patients admitted to the hospital whether through the Emergency Department or an admission unit. The protocol and data extraction was conducted according to the 2009 PRISMA guidelines[5], the completed checklist is available from the authors upon request. We defined the relevant outcome to be either in-hospital mortality (at any point in time) or transfer to the ICU. Only studies validating variables using a relevant scientific principle (regression analysis, discriminate analysis, recursive partitioning analysis or neural network) and not derived for only a single or a few diagnoses (e.g. only critically ill patients admitted to the ICU or patients admitted with sepsis) were included. The system had to be practicable without requiring extensive computer resources.

Thus, we conducted a search of PubMed (1950 till 2008 week 38) using the MeSH Terms ("Intensive Care Unit" OR "Mortality") AND "Health Status Indicators" AND ("Patient admission" OR "Hospitalization"). We also searched EMBASE (from 1980 till 2008 week 38) using the terms (including related terms) ("Mortality" OR "Intensive Care Unit") AND "Scoring system". A search of The Cochrane Library using the term "Scoring system" was also conducted.

We included literature from our own archives, and a hand-search was conducted in every selected article for relevant references for inclusion.

The results of searches were analyzed independently by two authors (MB and NGC). Data were extracted by two authors (MB and LF) and disagreement was resolved by consensus.

Whenever possible, we extracted the scoring system's discriminatory power (i.e. the ability to identify patients at increased risk of meeting the outcome), expressed as the area under receiver operating characteristic curve (AUROC)[6] and its calibration (i.e. agreement between the predicted and the observed outcome in the model), expressed as the p-value of the Hosmer-Lemeshow goodness-of-fit test[7]. AUROC values above 0.8 were considered as reflecting good discriminatory power.

In addition, we classified the scoring system's level of evidence by the method suggested by McGinn et al[8] and ascertained whether the system had been applied on a new cohort of patients, either at the same location or at external location (i.e. internal or external validation), in order to assure that the system is applicable to other groups than those on which is was developed.

## Results

The PubMed search resulted in 1,587 hits, EMBASE in 68 and the Cochrane Library none.

Thirty articles were selected for further review. Two were excluded due to use of endpoints other than those specified in the search strategy, one was a narrative review, four were conducted in other environments than the specified, one was a consensus paper, six were for specified groups of patients and one was not a scoring system.

Six articles were on track and trigger systems (scoring systems normally used to activate in-hospital Medical Emergency Teams to evaluate patients in acute distress). One was a review of 33 different systems and this was included in our review. Two of these six articles included only patients who presented to an Emergency Department and were thus excluded as the patients were not later admitted to the hospital.

**Figure 1:**
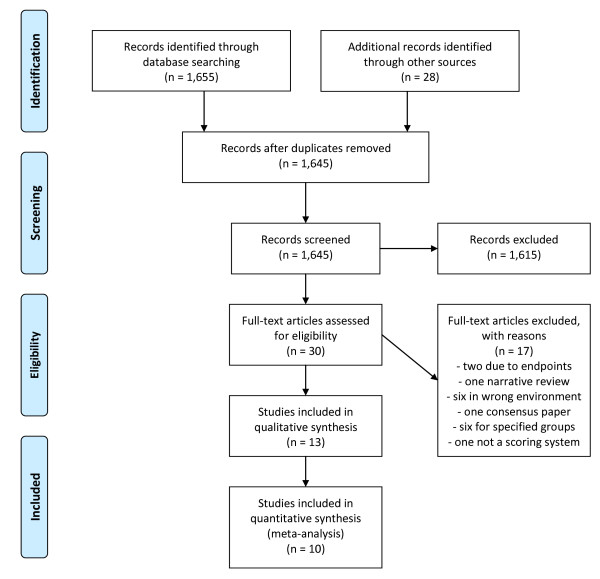
**Search strategy used for this article according to the PRISMA guidelines**.

A total of 13 articles were included in this review, see figure 1.

The articles presented in this study are very heterogeneous. They originate from different department types and the case-mix ranges from patients solely admitted by helicopter to all patients discharged from a medical department. We therefore have chosen to focus only the parts of the scoring systems we find important for assessing their relevancy; i.e. which variables have the authors chosen to include, which statistical methods were chosen to design and test the systems, what was the discriminatory power and calibration of the systems (i.e. how usable are the systems) and which level of evidence does the systems achieve.

### Track and trigger systems

Two of the papers on track and trigger systems were written by Subbe et al. One analyzed the Early Warning Score (EWS)[9] and the other the Modified Early Warning Score (MEWS)[10] on patients admitted to or through a MAU. None of these articles presented data on discriminatory power or calibration. When calculating the EWS, the authors found that a maximum score of five was associated with an increased risk of death, ICU and HDU admission[9]. When the authors stratified patients into three risk bands according to the MEWS score, they only found a statistical significant increased incidence of cardiac arrest in the intermediary risk band i.e. MEWS 3-4[10].

Paterson et al. included medical and surgical patients admitted to a combined assessment area in their study[11]. The object was to evaluate the implementation of a standardized early warning scoring system (SEWS). A total of 848 patients were included, 435 after the implementation of SEWS. In the SEWS cohort, they found a significant linear relationship between in-hospital mortality and admission SEWS score (chi-squared 34.3, p < 0.001). Data on discrimination were not presented.

As the review by Smith et al. is included in our article (here referred to as TTS) and included the studies by both Subbe et al. and Paterson et al., these will not be presented in further detail.

### Variables included in the scoring systems

All but two of the scoring systems used vital signs as variables when calculating the score (see table 1). *The Admission Laboratory Tests *(ALT)[12] and *The Routine Laboratory Data *(RLD)[13] both relied mostly upon blood tests. Two systems, the *Simple Clinical Score *(SCS)[14] and the *Hypotension, Oxygen saturation, low Temperature, ECG changes and Loss of independence Score *(HOTEL)[15] included both subjective and objective parameters (e.g. dyspnoea and abnormal EKG).

**Table 1:** Parameters included in the scoring systems

Parameter	**ALT**[12]	**EWS**[19]	**TTS**[16]	**RLD**[13]	**WPS**[20]	**SCS**[14]	**HOTEL**[15]	**RAPS**[17]	**REMS**[21]	**GS**[18]
Age	•			•		•			•	•
Sex				•						
Admission (acute or planned)				•						
Ability to stand unaided						•	•			
Coma						•				
New stroke						•				
Dyspnoea						•				
Nursing home resident						•				
Diabetes						•				
Abnormal EKG						•	•			
Spend time in bed prior to admission						•				

Vital signs										

Heart rate		•	•		•	•		•	•	
Systolic blood pressure		•	•		•	•	•			
Diastolic blood pressure			•							
Mean blood pressure								•	•	
Respiratory rate		•	•		•	•		•	•	
Temperature		•	•		•	•	•			
Level of consciousness (AVPU)		•	•^1^		•					
Level of consciousness (GCS)			•^1^					•	•	•
Oxygen saturation		•	•		•	•	•		•	•
Urine production			•^2^							
Altered mental state						•				

Blood tests										

Albumin	•			•						
Lactate dehydrogenase	•									
Alanine transaminase	•									
Aspartate aminotransferase	•									
Creatinine				•						
Blood Urea Nitrogen	•			•						
Sodium				•						
Potassium				•						
Glucose	•									
Haemoglobin				•						
Leucocytes	•			•						
Neutrophilocytes	•									

^1 ^Either AVPU or GCS according to original study, review article

^2 ^Regarded as normal in all cases (not analyzed in the study), review article

### Development of the scoring systems

Regression analysis was the most applied method for development of the scoring systems, only *Track and Trigger System *(TTS)[16] was developed otherwise (see table 2). Eight of the ten systems included patients admitted to a medical admission unit, but the population in *Rapid Acute Physiology Score *(RAPS)[17] was patients transported to the hospital by helicopter, and the population in the *Goodacre Score *(GS)[18] was patients transported to the emergency department by ambulance.

**Table 2:** Development data from the studies

Scoring System	Population	Exclusion criteria	Endpoint(s)	Number of endpoints met	Sex	Statistic method	Potential maximum population (before exclusion)	Sample-size (% of potential maximum population)	Number of parameters analyzed
REMS[21]	Patients admitted to a non-surgical emergency department	Cardiac arrest with unsuccessful resuscitation, more than one vital sign missing	In-hospital mortality	285 (2.4%)	51.6% female	Multivariate regression analysis	12,006	11,751 (97.9%)	8

RAPS[17]	Patients transported to a university hospital by helicopter	Age younger than 11 years, missing values	24 hours mortality	36 (12.7%)	NS	Multivariate regression analysis	283	283 (100%)	4

GS[18]	Patients transported to an emergency department by ambulance	Trauma, psychiatric disease, dead on arrival, discharged from the ED, outcome not available at follow-up, not admitted due to specified disease	In-hospital mortality	711 (12.7%)	42.3% female	Regression analysis	17,950	5,583 (31.1%)	3^1^

HOTEL[15]	Patients admitted to a medical admission unit	Age younger than 14, death < 15 minutes from arrival, missing values	Death within 15 minutes to 24 hours after arrival	59^2 ^(0.6%)	NS	Logistic regression	11,124	10,290^3 ^(92.5%)	11

SCS[14]	Patients admitted to a medical admission unit	Age younger than 14	30 days mortality	316 (4.7%)	47.5% female	Logistic regression	11,124	9,964^4 ^(89.6%)	29

WPS[20]	Patients admitted to an emergency care unit	None	In-hospital mortality	270 (8.5%)	52% female	Logistic regression	4,384	3,184 (72.6%)	6

RLD[13]	Patients discharged from medical department	Age below 16 at admission	In-hospital mortality	NS^5^	NS	Logistic regression	17,417	16,7377^6 ^(96.1%)	7

TTS[16]	Patients admitted to a medical admission unit	Age below 16, admission directly to the ICU	In-hospital mortality	835 (8.4%)	52.3% female	Comparison using AUROC	10,051	9,987 (99.4%)	8^7^

EWS[19]	Patients admitted to a medical admission unit	None	In-hospital mortality, length of stay, admission to ICU or CCU	29 combined (12.8%), 8 dead (3.5%)	48.5% female	Logistic regression	225	225 (100%)	6

ALT[12]	Patients in a medical emergency department	No blood test drawn	Mortality while admitted to a medical department	573 (5.6%)	48.6% female	Logistic regression	23,397	10,308 (44.1%)	27

NS = not specified

^1 ^saturation only available for 51.4% of the patients

^2 ^40 in the development cohort

^3 ^sample-size divided for validation purposes, 6,947 used for development

^4 ^sample-size divided for validation purposes, 6,736 used for development

^5 ^only specified for validation cohorts

^6 ^sample-size divided for validation purposes, 9,497 used for development

^7 ^either AVPU or GCS. Urine production was set to normal

Six systems used in-hospital mortality as their primary endpoint and only *Early Warning Score *(EWS)[19] used a composite endpoint.

### Discriminatory power and calibration

Discriminatory power (i.e. the ability to identify patients at increased risk of meeting the endpoint) was specified for eight of the scoring systems (see table 3), but not for RAPS and RLD. It was above 0.8 in five of these, but in *Worthing Physiological Scoring System *(WPS)[20] is was 0.74 and in TTS 0.657-0.782.

**Table 3:** Evidence level and validation of scoring systems to predict in-patient mortality in the medical admission unit

Scoring system	**Level of evidence**^1^	Validated in a new population at same site as developed	Validated in a new population at an external location	Discrimination (ability to identify patients at risk), AUROC	Calibration (agreement between predicted and observed risk), Chi-square
REMS[21]	3	•	•	0.852 (+/- 0.014)	487.3 (p < 0.0001)

RAPS[17]	3	-	•	NS	NS

GS[18]	4	-	-	0.81 (95% CI: 0.78-0.84)	NS

HOTEL[15]	3	•	-	0.865 (0.793-0.937) Validation: 0.854 (0.746-0.962)	1.49 (p = 0.83)

SCS[14]	3	•	-	0.858 (SE 0.009) Validation: 0.856 (SE 0.013)	NS

WPS[20]	3	•	-	0.74 (0.74-0.77)	p = 0.119

RLD[13]	3	•	-	^2^	^2^

TTS[16]	2	•	•^3^	0.657-0.782	NS

EWS[19]	2	•	-	0.68 (0.65-0.71)	NS

ALT[12]	3	•	-	0.904	^4^

NS = not specified

^1 ^according to McGinn et al[8]

^2 ^only specified for validation cohorts

^3 ^several publications validate TTS

^4 ^mentioned to be good, but not specified

The calibration (i.e. agreement between the predicted and the observed outcome in the model) was only specified for four scoring systems. In The *Rapid Emergency Medicine Score (REMS) *[21] it was calculated using the Chi-square test and was found to be poor, but not further specified.

In none of the studies impact analysis or inter-observer reliability were analyzed.

### Evidence level

The TTS and EWS reached evidence level two according to McGinn et al. The GS only reached level four whereas the other systems all reached level three. None of the systems thus reached level one.

## Discussion

We identified ten different scoring systems assessing the risk of in-hospital mortality or admission to an ICU in acutely admitted patients. None of these systems complied with the criteria for the highest levels of scientific evidence, but all seemed somewhat scientifically sound and could perhaps be used in a MAU. Most of the scoring systems use primarily vital signs as variables in the attempt to stratify the patients. The SCS and HOTEL scores use some subjective data (e.g. dyspnoea). The ALT and RLD use biochemical analyses and therefore cannot be calculated on presentation of the patient but have to await the analyses of blood tests. Data for calculating the other eight scores are easily obtained at presentation (except perhaps the EKG needed for calculation of SCS and HOTEL) and the score can be calculated at this early point in time. The WPS, EWS, TTS, SCS, GS, REMS and RAPS use an aggregate weighted score where increasing abnormality in the variables results in an increased score (e.g. respiratory rate ≤ 19 scores 0, 20-21 scores 1 and ≥ 22 scores 2). The RLD and ALT uses a mathematical formula to calculate the risk (e.g. -10.192 + (-0.013 * gender) + (5.717 * mode of admission) + (0.018 * urea) etc.). The HOTEL score simply adds one point to each of the criteria that are outside the defined interval (e.g. systolic blood pressure < 100).

The ability of the scoring system to separate the patients with increased risk for meeting the specified outcome (e.g. mortality) is determined by the discriminatory power. The RAPS, RLD and EWS however, do not present this in their article. As for the other seven scoring systems all have an AUROC above 0.657, indicating at least a fair discriminatory power. Both the HOTEL score and the SCS reach impressive AUROC's during both development and validation.

Calibration, i.e. the agreement between the predicted and the observed outcome across all patients stratified into subgroups, was not reported systematically. In fact, only four articles (REMS, HOTEL, WPS and partly ALT) presented data on this subject. In REMS the calibration was poor, but it was reported as satisfactory to good in the other studies.

A developed scoring system can only be used if it has been validated (i.e. applied to a new cohort of patients). Otherwise, the discriminatory power and calibration can be falsely elevated. There are several ways to validate a scoring system, but an external validation (i.e. at another location than were the system was developed) in a separate cohort is preferable. However, only three of the systems were validated externally (REMS, RAPS and TTS) and one scoring system was not even validated locally (GS). As described by McGinn et al[8] scoring systems can be categorized into levels of evidence according to their method of validation. The scoring systems in this study to reach the highest levels of evidence (level 2) were "Track and Trigger" systems, also used in activation of medical emergency teams. All other systems reached level 3 except the GS which only reached level 4 as it is not validated but only derived.

Most of the parameters used to calculate the scores are straight-forward, and the calculation does not seem complicated, perhaps with the exception of RLD and ALT which use a complicated formula derived from regression analyses. However, as none of the systems presents reliability data, it is unknown which level of inter-observer reliability is reached. In some of the scoring systems, a few parameters bear a risk of increased inter-observer variability, e.g. if the patients have dyspnoea (SCS), is able to stand unaided (SCS and HOTEL) and perhaps respiratory rate (EWS, TTS, WPS, SCS, RAPS and REMS).

But the main question is if we have any use for scoring systems in today's world of medicine. One thing is that scoring systems are capable of predicting mortality and ICU admissions, but does this have any clinical importance? Indeed one could argue that scoring systems bring little extra information to the clinical judgment made by all doctors on their first encounter with a patient. An example of this was the SUPPORT trial that showed that providing the physicians with objective outcome predictions, did not significantly change physician's attitudes and behavior[22,23] when treating their patients. In order to clarify this, we need studies comparing clinical assessment of patients with the combined effect of clinical assessment and the use of scoring systems. This has rarely been done in the Emergency Department, but we know from the critical care environment that physicians are good at prediction of mortality in patients by use of their clinical assessment, but that the use of scoring systems can support their judgment[1,24-26]. But even if it is eventually proven that scoring systems improve assessment of mortality, one could argue that the introduction of the scoring system itself forces the clinician to reflect on the risk of the patient, and that this carries all of the effect. However, the use of scoring systems will perhaps be able to identify patients at risk that might be overlooked by the medical staff and thereby improve their treatment, and this alone could justify their existence.

But most scoring systems are developed for use on groups of patients and not on an individual level. However, this fact is often overlooked by our inexperienced colleagues and the score is applied directly to the patient. This runs the risk of misclassifying the patient and thus directing therapy in, perhaps, a wrong direction. As none of the scoring systems presented in this paper have had an impact analysis performed, we do not know if their implementation will affect clinical therapy. If we are to use scoring systems as routine part of our clinical work, much more research is needed. As a result of this, we, at the moment, cannot use any of these systems on an individual level.

## Conclusion

When assessing acutely admitted medical patients as a young and inexperienced doctor, the use of scoring systems can help identifying patients at risk. We have identified ten different systems, most of which rely on vital signs in prognosticating the patients.

None of the systems identified reached the highest level of evidence as defined by McGinn et al[8]. However, both the HOTEL score and the SCS were impressive in both discriminatory power and calibration. The REMS showed an acceptable discriminatory power but poor calibration. As for calculation, the ALT and RLD may prove difficult to use, especially compared to the other scoring systems.

None of the scoring systems presented in this article are perfect and all have their weaknesses. As such, more research is needed (especially external validation and impact analyses) before the use of scoring systems can be fully implemented to the risk assessment of acutely admitted medical patients.

## Competing interests

The authors declare that they have no competing interests.

## Authors' contributions

NGC and MB conducted the search for relevant articles. LF and MB extracted data from the articles. All authors participated in drafting, revising and finally approved the article.
